# Bioactive Pigments from Marine Bacteria: Applications and Physiological Roles

**DOI:** 10.1155/2011/670349

**Published:** 2011-09-26

**Authors:** Azamjon B. Soliev, Kakushi Hosokawa, Keiichi Enomoto

**Affiliations:** Department of Environmental Systems Engineering, Kochi University of Technology, 185 Miyanokuchi, Tosayamada, Kami, Kochi 782-8502, Japan

## Abstract

Research into natural products from the marine environment, including microorganisms, has rapidly increased over the past two decades. Despite the enormous difficulty in isolating and harvesting marine bacteria, microbial metabolites are increasingly attractive to science because of their broad-ranging pharmacological activities, especially those with unique color pigments. This current review paper gives an overview of the pigmented natural compounds isolated from bacteria of marine origin, based on accumulated data in the literature. We review the biological activities of marine compounds, including recent advances in the study of pharmacological effects and other commercial applications, in addition to the biosynthesis and physiological roles of associated pigments. Chemical structures of the bioactive compounds discussed are also presented.

## 1. Introduction

### 1.1. Marine Bacteria and Its Role in Life Sciences

A wide variety of diseases and medical problems represent a challenging threat to humans, who since ancient times have searched for natural compounds from plants, animals, and other sources to treat them. Although the process of finding effective treatments against fatal diseases is difficult, extensive searches for natural bioactive compounds have previously yielded some successful results. The isolation and identification of specific natural compounds led to the development of folk medicine, and humans learned to separate the isolates into medicinal drugs, which could be used to treat different diseases, and poisonous substances, which could be used for nonmedicinal purposes (i.e., during tribal wars, hunting, etc.). Statistically, at least 50% of the existing drugs that are used to treat human illnesses are derived from natural products, most of which are obtained from terrestrial organisms [1]. However, due to continuous and exhaustive research, land-based natural bioactive compounds have become increasingly difficult to find. Instead, water-based natural compounds have become a more promising source, not only from a pharmacological view, but also for industrial and commercial applications. 

Theoretically, life is considered to have originated in the sea and, as a result of evolutionary changes, developed into a wide variety of diverse biological systems. The Earth's surface consists of 70% water, which is inhabited by 80% of all life forms [1], and consequently aquatic organisms have a greater diversity than their terrestrial counterparts. As research into the marine environment is still in its early phases, many mysteries associated with aquatic fauna and flora have yet to be discovered. Therefore, the marine environment has recently become an attractive research subject for many investigations, because of its rich biodiversity. Despite being comprised of a diverse ecosystem, the search for marine metabolites is difficult because of the inaccessibility and nonculturability of the majority of organisms [2]. Nevertheless, the existing technologies like deep seawater pumping facilities, scuba diving, and other available equipments, have facilitated investigation of the sea environment. As a result, scientific research has increasingly focused on marine biochemistry, microbiology, and biotechnology.

Microorganisms and their isolates represent a major source of undiscovered scientific potential. It should be noted that the number of microbial organisms isolated from the vast ocean territories continues to increase each year. Consequently, natural products isolated from microorganisms inhabiting environments other than soil are an attractive research tool, not only for biochemists and microbiologists, but also for pharmacologists and clinicians. Laatsch [3] described the isolation and description of nearly 250 marine bacterial metabolites versus 150 isolated from terrestrial bacteria between 2000 and 2005. Research into marine microorganisms and their metabolites has therefore become a major task in the search for novel pharmaceuticals.

Although many compounds show promising biological activities, it is difficult to point out any particular bioactive agent that has readily been commercialized as a medicine. Currently, 13 natural products isolated from marine microorganisms are being tested in different phases of clinical trials, and a large number of others are in preclinical investigations [4], thus highlighting the potential of marine natural compounds.

Despite thousands of marine bioactive compounds having been isolated and identified, in this paper, we will focus on the pharmacologically active pigmented compounds produced by marine microorganisms exhibiting *in vitro* or *in vivo* biological activities. Although pigmented compounds produced by terrestrial bacteria are beyond the scope of this review, specific examples will still be mentioned for comparative purposes, to outline common biological activities or because identical pigments were isolated from both types of microorganisms.

### 1.2. Marine Microorganisms and Their Bioactive Isolates

Marine and terrestrial microfloras differ from each other due to the influence of their respective environmental conditions. Microorganisms living in the sea must be able to survive and grow in the water environment with low nutrition, high salinity, and high pressure. That is why most bacteria isolated from seawater are Gram-negative rods, as it is postulated that their outer membrane structure is evolutionarily adapted to aquatic environmental factors. Marine microorganisms can be divided on the basis of habitat into psychrophiles (living at low temperatures), halophiles (living at high salinity), and barophiles (living under high pressure). Although these characteristics highlight the differences between marine and terrestrial microorganisms, it remains difficult to separate bacterial genera on the basis of habitat due to the ubiquitous presence of similar species in both environments. As such, most bioactive compounds have been isolated from bacteria in both environments. 

Marine bacteria, however, are attractive to researchers because they can potentially produce compounds with unique biological properties [5]. Until now, marine *Streptomyces, Pseudomonas, Pseudoalteromonas, Bacillus, Vibrio*, and *Cytophaga* isolated from seawater, sediments, algae, and marine invertebrates are known to produce bioactive agents. They are able to produce indole derivatives (quinones and violacein), alkaloids (prodiginines and tambjamines), polyenes, macrolides, peptides, and terpenoids. Examples of bioactive-pigmented compounds isolated from marine (and some terrestrial) bacteria are discussed below.

## 2. Pigments from Marine Bacteria

Bioactive pigments from marine bacteria are summarized in Table 1.

### 2.1. Prodiginines

**Figure 1:**
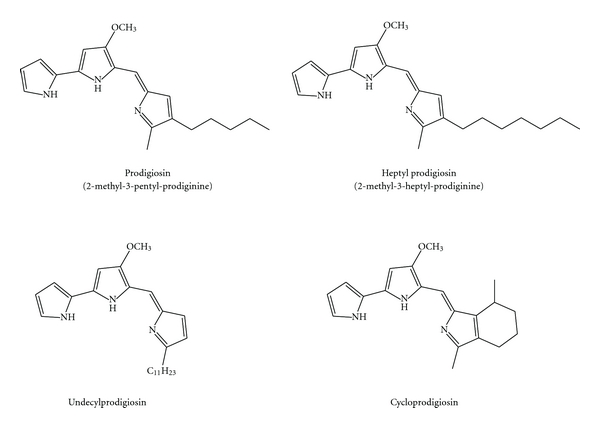
Prodiginine derivatives.

Red-pigmented prodigiosin compounds were first isolated from the ubiquitous bacterium *Serratia marcescens* and identified as secondary metabolites. The common aromatic chemical structure of these pigmented compounds was first named prodiginine by Gerber [6] (Figure 1). Prodigiosin was the first prodiginine for which the chemical structure was determined [7]. The name “prodigiosin” has been attributed to the isolation of prodigiosin from *Bacillus prodigiosus* bacterium (later renamed *Serratia marcescens*) [8], which was historically famed for the mysterious “bleeding bread” report [9, 10]. Prodiginines share a common pyrrolyldipyrromethene core structure and have a wide variety of biological properties, including antibacterial, antifungal, antimalarial, antibiotic, immunosuppressive, and anticancer activities [9, 11]. Such properties potentially make them one of the most powerful research tools in the past decade. 

There are many research reports and reviews regarding prodiginines and their biological activity investigations. In addition to the *Serratia*, several species of marine bacteria of the genera *Streptomyces* [8], *Actinomadura* [8], *Pseudomonas* [12], *Pseudoalteromonas *[13–18], and others [19] have also been reported to produce prodigiosin and related compounds. In particular, *Alteromonas denitrificans, *which was isolated from the fjord systems off the west coast of Norway [16] and later reclassified as *Pseudoalteromonas denitrificans* [20], has been reported to produce cycloprodigiosin. This compound has immunosuppressive, antimalarial, and apoptosis-inducing activities [18, 21, 22]. *Pseudoalteromonas rubra*, found in the Mediterranean coastal waters [13], also produces cycloprodigiosin, in addition to prodigiosins [14, 15]. **α*-Proteobacteria* isolated from a marine tunicate collected in Zamboanga, Philippines, was reported to produce heptyl prodigiosin. *In vitro* antimalarial activity against *Plasmodium falciparum* 3D7 (IC_50_ = 0.068 mM and SI = 20) was about 20 times the *in vitro* cytotoxic activity against L5178Y mouse lymphocytes [23]. *In vivo* experiments using *Plasmodium berghei*-infected mice, at concentrations of 5 mg/kg and 20 mg/kg, significantly increased their survival, while also causing sclerotic lesions at the site of injection. 

Other bacteria reported to produce red pigments include *Hahella* [24], *Vibrio* [25], *Zooshikella* [26], and *Pseudoalteromonas* [17], isolated from the coasts of Korea, Taiwan, and Japan. Kim et al. [27] identified red-pigmented prodiginines from *Hahella chejuensis*. Nakashima et al. also evaluated the biological activity of similar prodiginines from a bacterium assumed to belong to the genus *Hahella* [28]. Red pigment-producing bacterial species have further been isolated from river water [29, 30] and even from a swimming pool [31]. The most active prodiginine derivatives have already entered clinical trials as potential drugs against different cancer types [9].

Japan is surrounded by sea and has a bordering coastline of the Pacific Ocean in the South and the Sea of Japan in the North and West, and is consequently rich in marine resources. Therefore, one of the main tasks of our research group is to investigate the marine environment and its biodiversity, especially marine microorganisms and their respective metabolites.

Previously, a total of 85 strains of bacteria were isolated by our research group from the Pacific Ocean at a depth of 320 m off Cape Muroto in the Kochi Prefecture of Japan. Among them, 13 strains were found to produce a purple pigment and one a red pigment. The red pigment-producing bacterium was later named strain 1020R [32]. Detailed investigations have revealed that this strain is closely related to the prodigiosin-producing bacterium *Pseudoalteromonas rubra* and is Gram-negative with rod-shaped morphology. Physicochemical investigations have revealed that the pigment produced by this strain contains at least seven structurally similar prodiginine compounds. Chemical structures for four of these were successfully determined, and each only differed by the length of the alkyl chain attached to the C-3 position of the C-ring. These compounds were further identified as prodigiosin and its analogues 2-methyl-3-butyl-prodiginine, 2-methyl-3-pentyl-prodiginine (prodigiosin), 2-methyl-3-hexyl-prodiginine, and 2-methyl-3-heptyl-prodiginine. Compound cytotoxicity to U937 leukemia cells was strongly dependent on the length of these alkyl side chains, which decreased with an increase in chain length. 2-methyl-3-butyl-prodiginine was the most potent cytotoxic pigment among them. Molecular investigations into the cytotoxic mechanisms of these prodiginine derivatives demonstrated effects on caspase-3 activation and DNA fragmentation, indicating the potential to induce apoptosis in leukemia cells.

### 2.2. Carotenes

Carotenes are polyunsaturated hydrocarbons that contain 40 carbon atoms per molecule and are exclusively synthesized by plants. They are orange photosynthetic pigments important for plant photosynthesis. Recently, an unusual halophilic bacterium, which requires 15–25% salt for its normal growth, was found in Santa Pola near Alicante and on the Balearic island of Mallorca, Spain. It appeared to be red or pink due to a wide variety of isoprenoid compounds (phytoene, phytofluene, lycopene, and *β*-carotene) produced by this prokaryote. Oren and Rodríguez-Valera [33] investigated red-pigmented saltern crystallizer ponds in these areas of Spain and demonstrated that the pigments were carotenoid or carotenoid-like compounds produced by halophilic bacteria related to the *Cytophaga-Flavobacterium-Bacteroides* group. Thus, it has been shown that *Salinibacter* is an important component of the microbial community that contributes to the red coloration of Spanish saltern ponds. 

**Figure 2:**
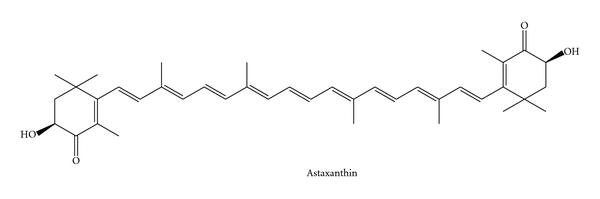
Astaxanthin.

Astaxanthin is one of the carotenoids that have commercial value as a food supplement for humans and as food additives for animals and fish (Figure 2). A carotenoid biosynthesis gene cluster for the production of astaxanthin has been isolated from the marine bacterium *Agrobacterium aurantiacum* [34]. Recently, another astaxanthin-producing marine bacterium was isolated and identified as *Paracoccus haeundaensis* [35].

### 2.3. Violacein

**Figure 3:**
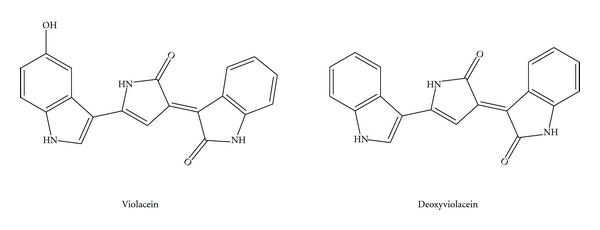
Violacein and deoxyviolacein.

The violet pigment violacein is an indole derivative, predominantly isolated from bacteria of the genus *Chromobacterium* that inhabit the soil and water of tropical and subtropical areas [36]. Over the past decade, the biosynthesis and biological activities of violacein have been extensively studied, and many scientific papers and reviews have been published [37–41]. Violacein has a variety of biological activities, including antiviral, antibacterial, antiulcerogenic, antileishmanial, and anticancer properties [36, 37, 41, 42] (Figure 3). Use of violacein as a chemical defense against eukaryotic predators has also been investigated [43–46]. 

One of the first published reports on violacein production by marine bacteria was by Hamilton and Austin [47]. This bacterial strain, *Chromobacterium marinum, *was isolated from open ocean waters and produced a blue pigment that was identified as violacein on the basis of physicochemical characteristics [47]. Later, Gauthier [48] described 16 violet-pigmented heterotrophic bacilli isolated from Mediterranean coastal waters and proposed the name *Alteromonas luteo-violaceus* for these strains. Another six bacterial species were also isolated by Gauthier et al. [49] from neritic waters on the French Mediterranean coast and were very similar to *Alteromonas* species. These species produced characteristic pigmentations ranging from pinkish-beige with reddish-brown diffusible pigment, lemon yellow, bright red turning carmine in old cultures, and orange to greenish-brown. Light violet, dark violet, or almost black pigments were also produced and later identified as violacein. The strains showed antibiotic activity against *Staphylococcus aureus* [49]. Subsequently, many other reports on violacein production have been published [50, 51]. 

Several purple pigment-producing *Alteromonas *species were also isolated from Kinko Bay in Kagoshima Prefecture, Japan. One of these, *Alteromonas luteoviolacea* (reclassified as *Pseudoalteromonas luteoviolacea*), is the only extensively characterized marine bacterium ever reported that produces violacein [48, 52, 53]. Previously, we have also reported 13 strains of Gram-negative, rod-shaped bacteria that produce a violacein-like purple pigment, which were isolated from the Pacific Ocean at a depth of 320 m off the coast of Cape Muroto, Kochi Prefecture, Japan [32]. Among them, two groups of novel violacein and deoxyviolacein producing marine bacteria were isolated and characterized in detail [50]. Biological investigations of violacein produced by these strains revealed potent cytotoxic effects against U937 and HL60 leukemia cell lines, with an IC_50_ value of 0.5–1 *μ*M. The molecular mechanisms currently known to be involved in violacein cytotoxicity include caspases activation, chromatin condensation, and DNA fragmentation, which all contribute to cell apoptosis. Recently, we also demonstrated that the protein kinases actively involved in the signal transduction pathway are also targeted by violacein.

### 2.4. Phenazine Compounds

**Figure 4:**
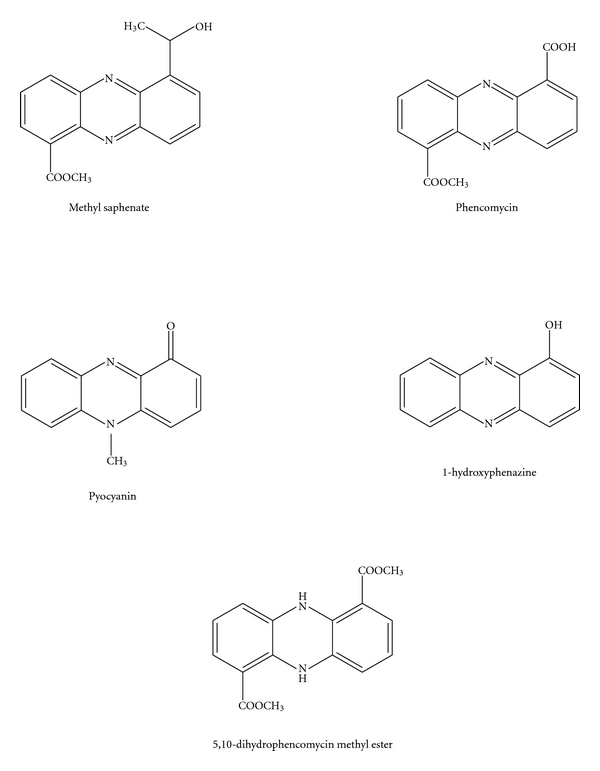
Phenazine derivatives.

Phenazines are redox-active, small nitrogen-containing aromatic compounds produced by a diverse range of bacterial genera, including *Streptomyces* (terrestrial), *Pseudomonas *(ubiquitous),* Actinomycetes* (terrestrial and aquatic), *Pelagibacter *(aquatic), and *Vibrio *(aquatic), under the control of quorum sensing [54, 55] (Figure 4). These compounds were subjected to extensive studies due to their broad spectrum of antibiotic activities against other bacteria, fungi, or plant/animal tissues [56–62]. Phenazine color intensity may vary among the derivatives and range from blue, green, purple, yellow, red to even brown [58, 63]. More than 6,000 phenazine derivatives have been identified and described during the last two centuries [59]. 

Maskey et al. [63] reported the isolation of two yellow pigments from the marine *Pseudonocardia *sp. B6273, a member of the *Actinomycetes.* Structural investigations identified the two pigments as novel phenazostatin D, inactive against the tested microorganisms, and methyl saphenate, a known phenazine antibiotic. Li et al. [64] also reported the isolation of a novel phenazine derivative with cytotoxic effects against P388 cells, together with six previously identified compounds from the marine *Bacillus *sp., collected from a Pacific deep-sea sediment sample at a depth of 5059 m. A novel phenazine derivative with antibiotic activity, identified as 5,10-dihydrophencomycin methyl ester, along with (2-hydroxyphenyl)-acetamide, menaquinone MK9 (II, III, VIII, IX-H8), and phencomycin, was isolated from an unidentified marine *Streptomyces *sp. by Pusecker et al. [65].

Pyocyanin and 1-hydroxyphenazine also downregulate the ciliary beat frequency of respiratory epithelial cells by reducing cAMP and ATP, alter the calcium concentration by inhibition of plasma membrane Ca^2+^-ATPase, and induce death in human neutrophils [60, 61, 66]. Due to the abundance and biotechnological application of *Pseudomonas aeruginosa* phenazines, pyocyanin and pyorubrin have also been suggested as food colorant pigments [58].

### 2.5. Quinones

**Figure 5:**
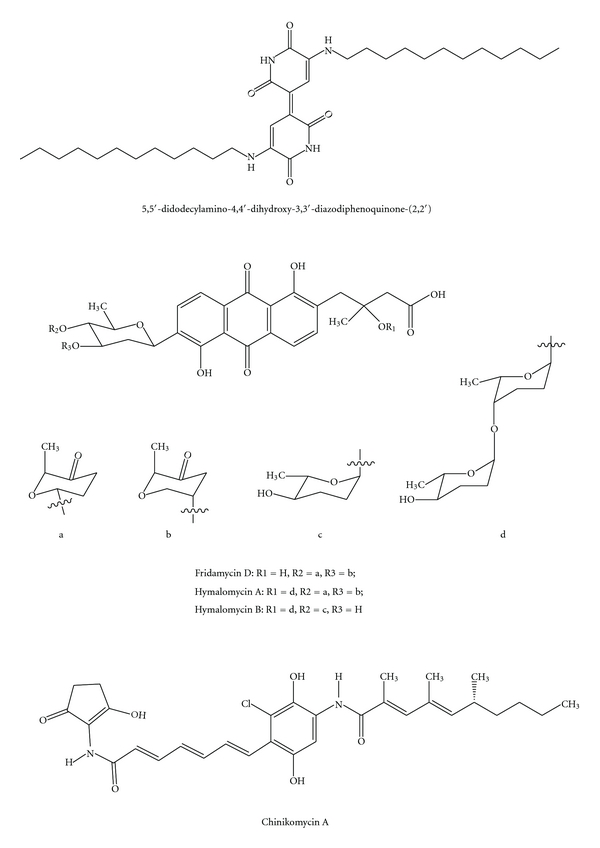
Quinones.

Quinones are additional colored compounds with an aromatic ring structure that have been isolated from marine environment [67, 68] (Figure 5). Quinone derivatives range in color from yellow to red, exhibit antiviral, anti-infective, antimicrobial, insecticidal, and anticancer activities, and have many commercial applications as natural and artificial dyes and pigments [69, 70]. 

*Streptomyces *sp. B6921 strain produced glycosylated pigmented anthracycline antibiotics, including fridamycin D and two new compounds, named himalomycin A and B, each of which displayed similar levels of strong antibacterial activity against *Bacillus subtilis*, *Streptomyces viridochromogenes *(Tü 57), *S. aureus*, and *Escherichia coli*. This strain also produced rabelomycin, *N*-benzylacetamide, and *N*-(2′-phenylethyl) acetamide [68]. Two novel pigmented antitumor antibiotics, chinikomycin A and B, together with manumycin A, were isolated from a marine *Streptomyces *sp. strain M045 [71]. The two chlorine containing quinone derivatives were shown not to have antiviral, antimicrobial, and phytotoxic activities; however, they exhibited antitumor activity against different human cancer cell lines. Chinikomycin A selectively inhibited the proliferation of mammary cancer, melanoma, and renal cancer cell lines, while chinikomycin B showed selective antitumor activity against a mammary cancer cell line [71]. 

Other bacteria, including a marine isolate *Pseudomonas nigrifaciens* (later reclassified as *Alteromonas nigrifaciens*), produce the blue pigment indigoidine [72]. Kobayashi et al. [73] isolated a new violet pigment with an alkylated indigoidine structure from *Shewanella violacea, *a deep-sea bacterium from sediments of Ryukyu Trench at a depth of 5110 m. This pigment was established as 5,5′-didodecylamino-4,4′-dihydroxy-3,3′-diazodiphenoquinone-(2,2′) based on X-ray diffraction analysis of single crystals. It does not have antibiotic activity against *E. coli*; however, it could potentially be used as a dye because of its high stability and low solubility. Thus, it could be suitable for industrial applications.

### 2.6. Tambjamines

It has long been noticed that marine bacteria have the ability to prevent biofouling. Holmström et al. [74] found that, amongst the marine *Pseudoalteromonas* species, *P. tunicata *has the widest range of antibiofouling activities against microorganisms, including bacteria, invertebrate larvae, algal spores, protozoan, and fungi, and provides protection for host marine organisms. These activities were linked to the production of unidentified yellow and purple pigments [75]. Recently, this yellow pigment was isolated from *P. tunicata *and was identified as a new member of the tambjamine class of compounds [76]. 

**Figure 6:**
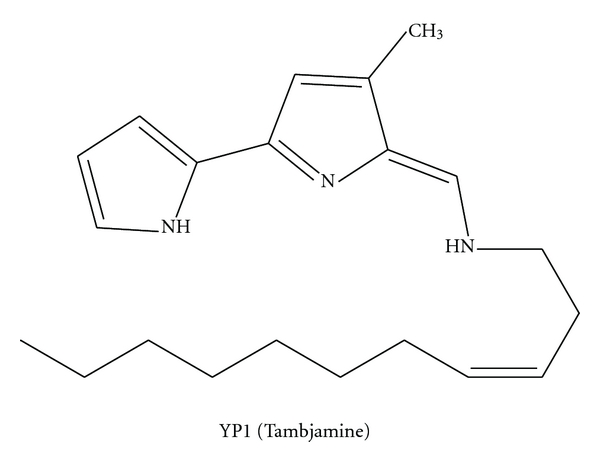
Tambjamine.

Tambjamines (Figure 6) are alkaloids isolated from various marine organisms like bryozoans, nudibranchs, and ascidians [77–79]. This yellow pigment has also been isolated from marine bacteria [76]. The tambjamines also exhibit antibiotic activity against *E. coli*, *Staphylococcus*, *Vibrio anguillarum* [77], *B. subtilis*, and *Candida albicans *[80, 81] and displayed cytotoxic activity against several tumor cell lines [80]. Recently, Pinkerton et al. [80, 82] reported the first total synthesis of nine tambjamines and their antimicrobial and cytotoxic activities. All of the tested tambjamines showed antibacterial, antifungal, and cytotoxic effects that contributed to cell death through apoptosis, but not necrosis. These activities were, however, lesser than the positive control (doxorubicin) [80].

### 2.7. Melanins

*Vibrio cholerae, Shewanella colwelliana*, and *Alteromonas nigrifaciens* were some of the first marine bacterial strains described to produce melanin or melanin-like pigments [83–86]. The pigment synthesized by *Vibrio cholerae* was reported to be a type of allomelanin derived from homogentisic acid [87]. Melanin formation in *V. cholerae* is a consequence of alterations in tyrosine catabolism and not from the tyrosinase-catalyzed melanin synthetic pathway. *Cellulophaga tyrosinoxydans* was reported to have tyrosinase activity and produce a yellow pigment suggested to be a pheomelanin [88].

The most illustrative example of melanin-producing marine bacteria is the actinomycetes. This is particularly the case for the genus *Streptomyces, *from which most compounds with known biological activity have been isolated [89]. All* Streptomyces *strains are reported to use tyrosinases in the synthesis of melanin pigments [90]. Another important melanin-synthesizing bacterium is *Marinomonas mediterranea*, which produces black eumelanin from L-tyrosine [91].

### 2.8. Other Pigmented Compounds

**Figure 7:**
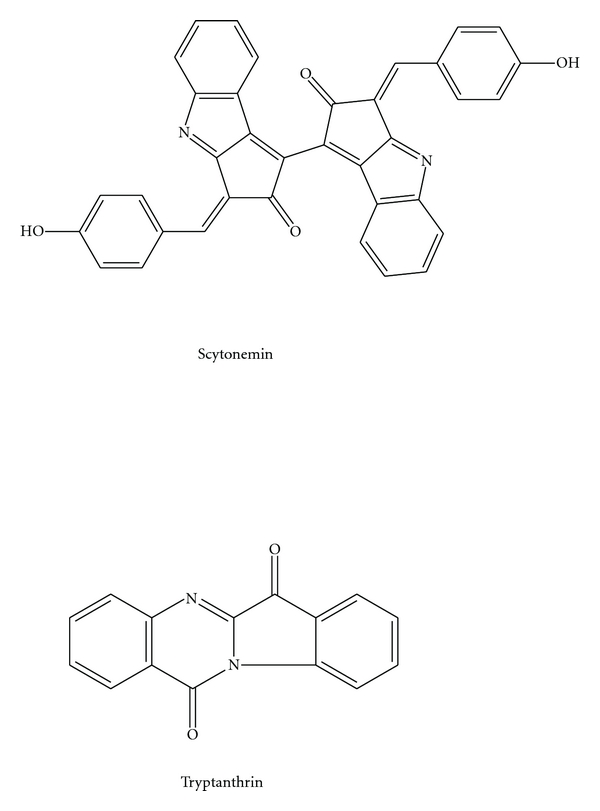
Other pigmented compounds.

Scytonemin, a yellow-green pigment isolated from aquatic cyanobacteria, forms when the bacteria are exposed to sunlight (Figure 7). It protects bacteria by preventing about 85–90% of all UV-light from entering through the cell membrane [92]. High UV-A irradiation inhibited photosynthesis and delayed cellular growth until sufficient amounts of scytonemin had been produced by the cyanobacteria. Scytonemin may also have anti-inflammatory and antiproliferative activities by inhibiting protein kinase C*β* (PKC*β*), a well-known mediator of the inflammatory process, and polo-like protein kinase 1 (PLK1), a regulator of cell cycle progression [93]. In addition, scytonemin inhibited phorbol-induced mouse ear edema and the proliferation of human umbilical vein endothelial cells.

Recently, two **γ*-Proteobacteria* strains of the genus *Rheinheimera* were isolated from the German Wadden Sea and from Øresund, Denmark that produced a deep blue pigment [94]. Structural analysis of the pigment revealed that this new compound has no similarity with any known blue pigments, like violacein and its derivatives. Due to its blue color and marine origin, the new pigment was named glaukothalin (from Greek *glaukos* “blue” and *thalatta* “sea”). The ecological role and biological activities of glaukothalin are currently under investigation.

AM13,1 strain, which was identified to belong to the *Cytophaga/Flexibacteria* cluster of North Sea bacteria, was found to produce yellow tryptanthrin, a rare compound that had never before been found in bacteria [95]. This compound was suggested to be a biocondensation product of anthranilic acid and isatin and exhibited a broad yet moderate antibiotic activity. Thus, the yellow color of the AM13,1 colonies was potentially due to their tryptanthrin content. In another yellow cultured Hel21 strain, pigment color may be a consequence of carotenoid zeaxanthin or one of the many vitamin K derivatives (e.g., menaquinone MK6) [95].

## 3. Biosynthesis of Pigments

Numerous reports detail the regulation and biosynthesis of bacterial secondary metabolites. Increased research and verification of specific bacterial pathways has predominantly been due to the antibiotic, immunosuppressive, and anticancer potential of these compounds. A brief discussion of this topic is given next, as detailed information is further provided in the cited references.

Biosynthesis of bacterial prodiginines has extensively been studied and reviewed [96, 97]. Prodigiosin biosynthesis was proposed to originate during the enzymatic condensation of 2-methyl-3-n-amyl-pyrrole (MAP) and 4-methoxy-2,2′-bipyrrole-5-carbaldehyde (MBC) precursors. Prodiginine biosynthetic gene clusters for *Serratia* sp. ATCC 39006 [98], *Serratia marcescens* ATCC 274 [98], *Hahella chejuensis* KCTC 2396 [27, 99], and *Streptomyces coelicolor* A3(2) [100] have been identified, sequenced, and expressed. Several gene clusters are involved in the biosynthetic pathway, depicted as *pig* in* Serratia* strains, *red* in *S. coelicolor* A3(2), and *hap* (numbered) in *H. chejuensis* KCTC 2396, with each encoding several proteins responsible for synthesis. The largest gene cluster found in *S. coelicolor* A3(2) consists of four transcriptional units, whereas the other three clusters are strongly homologous to each other and are arranged uni-directionally. 

In *Serratia* strains, *pig*B–*pig*E genes were identified to encode proteins responsible for the biosynthesis of MAP and condensation with MBC to form prodigiosin [96, 97]. A common pathway of MBC biosynthesis is proposed for all strains, in which proline, acetate, serine, and S-adenosylmethionine are incorporated into the bipyrrole at the initial stage [97]. PigA, PigF, PigG, PigH, PigI, PigJ, PigM, and PigN in* Serratia* strains and RedE, RedI, RedM, RedN, RedO, RedW, RedV, and RedX proteins in *S. coelicolor* A3(2) have been determined to participate in MBC biosynthesis [97]. PigB, PigD, and PigE enzymes in *Serratia* strains were proposed to be involved in the MAP biosynthesis, which requires 2-octenal as the initial precursor [97]. Monopyrroles condense with MBC during the final step of prodigiosin and/or undecylprodigiosin biosynthesis. PigC and its homologues catalyze this condensation in bacteria.

Some prodiginines can also be produced when monopyrroles are supplied to colorless *S. marcescens* mutants [8]. Addition of monopyrroles directly to a culture medium or as a vapor across the culture surface of a colorless mutant of *S. marcescens* resulted in the strain becoming initially pink and later red, indicating prodiginine formation [8]. Similar prodiginine biosynthesis produced by exogenously adding MAP and MBC was observed in white strains of *Serratia marcescens* isolated from patients [101].

The violacein biosynthesis pathway and associated biosynthetic enzymes have been extensively studied [38, 40, 102], although certain reactions and intermediates are yet to be elucidated. Currently, this proposed system involves an operon of five genes, *vioA*–*vioE*, which are transcriptionally regulated by a quorum-sensing mechanism that uses acyl-homoserine lactones as autoinducers. At the early stationary phase of bacterial growth, acylhomoserine lactones accumulate in the culture medium, inducing the transcription of the *vio* genes. Therefore, violacein is considered a typical secondary metabolite in bacteria. The first enzyme encoded by the *vio* gene operon, VioA, converts L-tryptophan to indole-3-pyruvic acid imine (IPA imine), and the second enzyme, VioB, catalyzes the reaction to convert IPA imine into an unidentified compound X (possibly an IPA imine dimer) [103, 104]. Compound X then undergoes successive reactions, catalyzed by the enzymes VioE, VioD, and VioC, to produce violacein.

Phenazine pigment biosynthesis reportedly involves shikimic acid as a precursor and forms chorismic acid as an intermediate product. Two molecules of chorismic acid then form phenazine-1,6-dicarboxylic acid, which is sequentially modified to create a variety of phenazine derivatives with different biological activities [105]. *Pseudomonas aeruginosa* PAO1 has two gene clusters (*phzA1B1C1D1E1F1G1* and *phzA2B2C2D2E2F2G2*), with each cluster capable of producing phenazine-1-carboxylic acid (PCA) from chorismic acid [106]. It is proposed that PhzM and PhzS catalyze the subsequent conversion of PCA to pyocyanin. In addition, PhzH is responsible for producing phenazine-1-carboxamide from PCA.

Fridamycin, hymalomycin, and chinikomycin are typical bacterial compounds that share a quinone skeleton. However, little information regarding the biosynthesis of these compounds has been accumulated.

Detection and identification of the entire *P. tunicata* gene cluster involved in the biosynthetic pathway production of the tambjamine YP1 using recombinant *E. coli *was conducted by Australian researchers Burke et al. [107]. In total, 19 proteins encoded the Tam cluster participate in the postulated biosynthetic pathway. Among them, 12 were found to have high sequence similarity to the red proteins responsible for undecylprodigiosin synthesis in *S. coelicolor* A3(2) and the pig proteins involved in prodigiosin biosynthesis in *Serratia* sp. [107]. Such similarity in the chemical structures of these two classes of compounds results in tambjamines having two pyrrole rings while the prodiginines have three. As is the case for the prodiginines, 4-methoxy-2,2-bipyrrole-5-carbaldehyde (MBC) is initially formed from proline, serine, and malonyl CoA in the tambjamine biosynthetic pathway. A double bond is inserted by TamT and an amino group is transferred by TamH to dodecenoic acid activated by AfaA, which is predicted to be an acyl-CoA synthase. The resulting dodec-3-en-1-amine is condensed with MBC by TamQ to form tambjamine YP1 [107].

In addition to* V. cholera*, *S. colwelliana*, *A. nigrifaciens*, and *C. tyrosinoxydans*, melanin syntheses have also been reported in *M. mediterranea*, which contains the tyrosinase gene operon [108], and in an epiphytic *Saccharophagus degradans* 2-40 bacterium [109]. While the specific details of melanin formation continue to be debated, well-defined biosynthetic schemes have now been proposed. Two different biosynthetic pathways synthesize the eumelanins and pheomelanins. Both pathways are initiated by the oxidation of L-tyrosine to 3,4-dihydroxyphenylalanine (DOPA) and the subsequent creation of dopaquinone by tyrosinase. The latter product is transformed either to pheomelanin by combining with cystein and forming an intermediate S-cysteinyldopa and benzothiazine or to eumelanin with intermediate leucodopachrome, dopachrome (red), 5,6-dihydroxyindole, 5,6-indolequinone (yellow) formation [69].

*Nostoc punctiforme *ATCC 29133 is the only scytonemin-producing organism whose genome has been fully sequenced [110]. This scytonemin biosynthesis potentially involves a gene cluster consisting of 18 open reading frames (ORFs) (NpR1276 to NpR1259). Although, the functional roles of all these ORFs are not yet fully determined, some intriguing hypotheses have been proposed. In particular, both tyrosine and tryptophan are implicated as biosynthetic precursors for scytonemin in the pigment formation pathway. NpR1275, which functionally resembles leucine dehydrogenase, is utilized in the early stages of scytonemin synthesis in *N. punctiforme*, thereby oxidizing tryptophan and/or tyrosine to their corresponding pyruvic acid derivative. 

Alternatively, it is suggested that NpR1269, a putative prephenate dehydrogenase, generates *p*-hydroxyphenylpyruvic acid, which is a derivative of tyrosine in the early pathway stages. NpR1276 uses two pyruvic acid derivatives from tryptophan and tyrosine for the synthesis of a labile *β*-ketoacid product, which is homologous to the thiamin diphosphate- (ThDP-) dependent enzyme acetolactate synthase. NpR1274 possibly catalyzes the intermediate cyclization and decarboxylation of the **β**-ketoacid product to form the indole-fused cyclopentane moiety of the pigment [111]. Monomer precursors that are formed then undergo dimerization to produce scytonemin. NpR1263, which was found to be similar to a tyrosinase in melanin biosynthesis, participates in these later oxidative dimerization steps, thereby forming scytonemin [112]. Functional roles of other ORFs and their putative intermediate products for the pigment production are still under investigation.

## 4. Concerns regarding the Physiological Role of Pigmented Compounds

A number of bacterial species, including those inhabiting the vast marine environment, produce a wide variety of pigments that are important to cellular physiology and survival. Many of these natural metabolites were found to have antibiotic, anticancer, and immunosuppressive activities. These secondary metabolites, produced by microorganisms mostly via the quorum sensing mechanism, have the ability to inhibit the growth of or even kill bacteria and other microorganisms at very low concentrations. Due to such diverse and promising activities against different kinds of diseases, these compounds can play an important role in both pharmaceutical and agricultural research. 

It still remains uncertain why these pigmented secondary metabolites from bacteria have antibiotic and/or cytotoxic activities. Although, their true physiological role is yet to be fully discovered, there are a few reports that provide reasonable explanations by making comparisons with nonpigmented bacteria. In particular, the relationships between pigment production and toxicity have been studied by Holmström et al. [113], who found that 90% of all dark-pigmented compounds taken from marine living surfaces showed inhibitory activity towards invertebrate larvae. Two fractions isolated after column chromatography, one colorless and the other a yellowish-green color, were identified as phenazine derivatives from unidentified marine *Streptomycete* sp. by Pusecker et al. [65]. The colorless fraction was biologically inactive, while the pigmented phenazine derivative showed highly active antibiotic properties. Previous studies have also demonstrated that marine bacterial metabolites with antibiotic properties were always pigmented [114]. Screening of 38 antibiotic-producing bacterial strains revealed that all pigmented bacteria belonging to the *Pseudomonas-Alteromonas* group displayed antibiotic activity, while nonpigmented bacteria were inactive.

Considering data from all reported literature, a number of reasonable biological functions for pigment production in bacteria have been established. In general, the pigmented marine isolates seem to play two important roles: firstly, they provide an adaption to environmental conditions, and, secondly, they provide defense against predators [115]. For instance, it has been shown that the brown colored melanin pigments produced by a variety of species, as well as a yellow-green colored scytonemin pigment isolated from cyanobacteria, protect cells from UV irradiation and desiccation [69, 93]. Therefore, in order to adapt to the excessive sunlight and survive under harmful UV irradiation, bacteria must produce these indispensable compounds. Griffiths et al. [116] found that carotenoids, which were later suggested to be a substitute for sterols, are an important structural component of microbial membranes [117] and may protect bacterial cells from photooxidation or damage caused by visible light irradiation. 

Several bacterial pigments that act as antagonists by exhibiting antibiotic activity against other organisms can be considered as potent weapons for survival and effective chemical defenses against eukaryotic predators. This class of bioactive agents includes almost all pigmented compounds commonly produced by *Pseudoalteromonas, Pseudomonas*, and *Streptomyces* species. These compounds inhibit the settlement of marine invertebrate larvae [118], the germination of algal spores [119] and protect the host surface by interfering with bacterial colonization and biofilm formation [74]. They may also inhibit other organisms that compete for space and nutrients. 

Such hypotheses are also supported by a number of studies that found that these bacterial compounds were active against other prokaryotes and even eukaryotes [120–128]. In many studies, pigmented bacterial strains demonstrated a strong and broad range of antibiotic activities against other organisms, while nonpigmented strains did not [74, 129]. A clear correlation between pigment production and antibacterial activities of the two *Silicibacter *sp. strain TM1040 and *Phaeobacter *strain 27-4 grown under static conditions was further reported by Bruhn et al. [129]. Mutant strains, which lacked pigment production, also lost their biological activities. Holmström et al. have also shown a close relationship between pigmentation and inhibitory activity, whereby 20 out of 22 dark pigmented bacterial strains tested displayed inhibitory activity against the settlement of two invertebrate larvae and algal spores [113].

Amongst other bacterial strains, *Pseudoalteromonas* has the most diverse antibiotic activities against alga biofouling, and the dark green pigmented *P. tunicata* exhibits the most active and broadest range of inhibitory activity when compared to other strains from this genus [74]. Two nonpigmented *P. nigrifaciens* and *P. haloplanktis* strains were also found not to display any antibiotic activities using various bioassays [74].

Blue-pigmented pyocyanin production in *P. aeruginosa* (Pup14B) was observed by Angell et al. to be induced by *Enterobacter* species (Pup14A and KM1), and this pyocyanin displayed moderate antibiotic activity against *E. coli* and yeast [130]. It was experimentally demonstrated that metabolites produced by Pup14A strain are necessary for the production of this pigment in Pup14B strain [130]. Many other reports describe synergism between bacteria and higher organisms; however, this is a rare example between two bacterial species [131]. Such an unusual case contrasts with the hypothesis of the regulated biodiversity of marine bacteria, in which surface-associated microorganisms produce antimicrobial agents [74] to prevent competing microorganisms. The symbiosis of the two bacterial species is not yet fully understood, although both species appear to benefit from the pigment production.

One of the promising biological activities of marine bacteria isolates is their cytotoxic effect against cancer cells. Despite many investigations, the exact molecular mechanism of this pigmented compound cytotoxicity remains undetermined and requires further study. For example, violacein is known to cause apoptosis in tumorous cells [41]. However, the pathways leading to cell death have not yet been linked to the possible effects of the pigment, which was also shown to affect signal transduction agents, such as protein kinase and protein phosphatase family enzymes that play crucial role in cell differentiation and proliferation. 

In a study by Bromberg et al., violacein showed inhibitory activity against protein phosphatases isolated from human lymphocytes [132]. A similar study was also conducted by Fürstner et al. to assess the inhibitory activity of prodigiosin derivatives [133]. Other targets of these compounds, including ion channels, are further being investigated [134–137].

Unexpected problems have also arisen when investigating marine environments. While the marine environment is a promising source for identifying microorganisms that can produce important biologically active pigments, yields of these compounds remain variable and are sometimes too low to provide enough material for drug development [138] or commercial applications. The main reason for such low yields is that these compounds are secondary metabolites and production depends on the quorum sensing mechanism.

Despite marine bacteria being capable of growing in the extremely low concentrations of nutrients that often exist in seawater, most species still require seawater or its equivalent as a growth medium for artificial culturing. Seawater is therefore used for the growth of marine bacteria, or similar levels of sodium, potassium, and magnesium chloride are supplemented in cultures. Optimal growth and the production of pigments are only sustained for most bacteria when appropriate salt mixtures are used for culturing, as is the case for the prodigiosin-producing marine *Pseudomonas magnesiorubra* and *Vibrio psychroerythrus*, among other marine species. These bacteria grew optimally and produced red pigment when cultured in seawater or its equivalent, while pigment production by the terrestrial *Serratia marcescens* was inhibited in 3% sea salts [8]. 

Enhancing low pigment productivity is one of the main issues facing researchers, and some solutions have already been reported. It is well established that antibiotic production by bacteria might be regulated both qualitatively and quantitatively by the nature of the culture medium. In particular, the addition of individual natural compounds to nutrient media or the use of gene expression methods was found to increase the pigment production far beyond expectations. For example, saturated fatty acids, especially peanut broth, was found to be a better choice in increasing prodigiosin production by 40-fold (approximately ~39 mg/mL) in *S. marcescens* [139]. 

Undecylprodigiosin synthesis by *S. marcescens* was also markedly enhanced by the addition of vegetable (soybean, olive, and sunflower) oils (2–6% [v/v]) and amino acids to the fermentation broth [140, 141]. Violacein production by the recombinant *Citrobacter freundii* strain, the genes of which were reconstructed from *Duganella* sp. B2, reached up to 1.68 g/L, making it fourfold higher than the highest production previously reported [142]. It is anticipated that these methods will facilitate the production of sufficient quantities of many bioactive and pharmacologically important compounds obtained from bacteria of marine origin. These compounds, including prodiginine and violacein, are now considered as potential drug candidates for potentially fatal diseases such as cancer and malaria. Although further improvement of culture methods and technologies for pigment production including recombinant technology is necessary, bioactive compounds from marine bacteria may potentially replace the existing drugs that have lower therapeutic actions.

## 5. Conclusions

Recently, a number of review papers have appeared in the literature, and they give an overview of all investigations of the marine environment and its isolates. While previous reviews have covered the biological activities of natural products isolated from marine microorganisms [115, 143] and other living organisms [144, 145], our paper is the first to review the importance of pigmented compounds from marine origin and their potential pharmacological applications.

Most studies investigating marine microorganisms have shown the efficacy and the potential clinical applications of pigmented secondary metabolites in treating several diseases. These studies have also emphasized the effects of microbial metabolites as antibiotic, anticancer, and immunosuppressive compounds. Despite the enormous difficulty in isolating and harvesting marine bacteria, significant progress has been achieved in this field, and investigations of bioactive compounds produced by these species are rapidly increasing. As such, the number of compounds isolated from marine microorganisms is increasing faster when compared with terrestrial species [95].

Overall, this review of pigmented marine bioactive compounds and their pharmacological applications highlights the importance of discovering novel marine bacterial metabolites. Such compounds have a wide variety of biologically active properties and continue to provide promising avenues for both fundamental sciences and applied biomedical research.

## Figures and Tables

**Table 1:** Biologically active pigmented compounds isolated from marine bacteria.

Pigment	Activity	Bacterial strains	References
(1) Undecylprodigiosin	Anticancer	*Streptomyces rubber*	[8]
(2) Cycloprodigiosin	Immunosuppressant; Anticancer; Antimalarial	*Pseudoalteromonas denitrificans*	[18, 21, 22]
(3) Heptyl prodigiosin	Antiplasmodial	**α*-Proteobacteria*	[23]
(4) Prodigiosin	Antibacterial; Anticancer; Algicidal	*Pseudoalteromonas rubra* *Hahella chejuensis*	[14] [27]
(5) Astaxanthin (carotene)	Antioxidation	*Agrobacterium aurantiacum*	[34]
(6) Violacein	Antibiotic; Antiprotozoan; Anticancer	*Pseudoalteromonas luteoviolacea * *Pseudoalteromonas tunicata * *Pseudoalteromonas* sp. 520P1 *Collimonas* CT	[48, 52, 53] [43] [50] [51]
(7) Methyl saphenate (phenazine derivative)	Antibiotic	*Pseudonocardia* sp. B6273	[63]
(8) Phenazine derivatives	Cytotoxic	*Bacillus* sp.	[64]
(9) Pyocyanin and pyorubrin	Antibacterial	*Pseudomonas aeruginosa*	[58]
(10) Phenazine-1-carboxylic acid	Antibiotic	*Pseudomonas aeruginosa*	[59]
(11) 5,10-dihydrophencomycin methyl ester	Antibiotic	*Streptomycete* sp.	[65]
(12) Fridamycin D, Himalomycin A, Himalomycin B	Antibacterial	*Streptomycete* sp. B6921	[68]
(13) Chinikomycin A and Chinikomycin B, Manumycin A	Anticancer	*Streptomycete* sp. M045	[71]
(14) Tambjamines (BE-18591, pyrrole and their synthetic analogs)	Antibiotic, Anticancer	*Pseudoalteromonas tunicata*	[76, 80]
(15) Melanins	Protection from UV irradiation	*Vibrio cholerae* *Shewanella colwelliana* *Alteromonas nigrifaciens* *Cellulophaga tyrosinoxydans*	[83, 84] [83, 86] [85] [88]
(16) Scytonemin	Protection from UV irradiation Anti-inflammatory, Antiproliferative	Cyanobacteria	[93]
(17) Tryptanthrin	Antibiotic	*Cytophaga/Flexibacteria* AM13,1 strain	[95]
